# Transcriptome-Wide Identification of miRNAs and Their Targets from *Typha angustifolia* by RNA-Seq and Their Response to Cadmium Stress

**DOI:** 10.1371/journal.pone.0125462

**Published:** 2015-04-29

**Authors:** Yingchun Xu, Lingling Chu, Qijiang Jin, Yanjie Wang, Xian Chen, Hui Zhao, Zeyun Xue

**Affiliations:** College of Horticulture, Nanjing Agricultural University, Nanjing, 210095, P.R. China; Kunming University of Science and Technology, CHINA

## Abstract

MicroRNAs (miRNAs) play important roles in plant responses to environmental stress. In this work, we used high-throughput sequencing to analyze transcriptome and small RNAs (sRNAs) in *Typha angustifolia *under cadmium (Cd) stress. 57,608,230 raw reads were obtained from deep sequencing of a pooled cDNA library. Sequence assembly and analysis yielded 102,473 unigenes. We subsequently sequenced two sRNA libraries from *T*. *angustifolia* with or without Cd exposure respectively. Based on transcriptome data of *T*. *angustifolia*, we catalogued and analyzed the sRNAs, resulting in the identification of 114 conserved miRNAs and 41 novel candidate miRNAs in both small RNA libraries. *In silico *analysis revealed 764 targets for 89 conserved miRNAs and 21 novel miRNAs. Statistical analysis on sequencing reads abundance and experimental validation revealed that 4 conserved and 6 novel miRNAs showed specific expression. Combined with function of target genes, these results suggested that miRNAs might play a role in plant Cd stress response. This study provided the first transcriptome-based analysis of miRNAs and their targets responsive to Cd stress in *T*. *angustifolia*, which provide a framework for further analysis of miRNAs and their role in regulating plant responses to Cd stress.

## Introduction

Toxic metal pollution of soils and waters, mainly caused by mining and burning of fossil fuels, is a major environmental problem facing the modern world. Cadmium (Cd), for example, which is a widespread toxic metal pollutant, is highly harmful to living organisms. Unlike organic pollutants, Cd cannot be chemically degraded or biodegraded by microorganisms. An alternative biological approach to deal with this problem is phytoremediation, i.e. the use of plants to clean up polluted waters and soils [1,2]. Phytoremediation is less expensive and particularly suitable for treatment of large volumes of substrate with low concentrations of toxic metals. However, the presence of toxic metals can lead to severe damage to plants including stunted plant growth [3–5], inhibition of photosynthetic activity [6] and decreased nutrient uptake [7]. In particular, Cd is a highly toxic and persistent environmental poison for plants and even animals. Free Cd in plasmatic compartments disturbs cell metabolism and regulation and results in serious plant injuries [4,5]. All these adverse effects of toxic metals limited the application of phytoremediation. Therefore, one trait of great significance to phytoremediation is the ability of plants to tolerate the toxic metals that are being extracted from the soil [1]. Thus, the elucidation of the regulatory mechanisms underlying toxic metal stress is becoming an urgent goal.

Gene expression is highly regulated in plants to ensure proper development and function of tissues and adequate responses to environmental changes including toxic metal stress [8]. A group of 21-24-nt small RNA molecules (sRNA) was recently found to be involved in regulating expression of genes [8,9]. These sRNAs are derived from double-stranded RNA, but formed through a different mechanism. MiRNA is one of the two main types of sRNAs, which are generated from non-coding transcripts with hairpin-structure by DICER-Like 1 (DCL1), which cleaves a short (21 bp) duplex from the stem region [10,11]. Mature miRNAs guide the RNA-induced silencing complex (RISC) to bind target genes through either cleaving target mRNAs or repressing their translation [12,13]. Besides numerous studies reported that miRNAs were involved in regulating a range of essential cellular and biological processes, increasing evidence showed that miRNAs also played crucial roles in plant responses to a variety of environment stresses, for example, drought [14], salinity [15], and toxic metal stress [16]. Recently, accumulating evidence revealed that miRNAs functioned as a key regulator in alleviation of plant metal stresses [17–20]. Several toxic metal responsive miRNAs have been reported and related regulatory mechanisms were detailed [18]. Thus, identifying and manipulating the expression of miRNAs involved in regulating toxic metals responsive targeting genes may be a good approach to enhancing toxic-metal tolerance in plant.

*Typha angustifolia* is a perennial herbaceous plant of genus *Typha*. *Typha angustifolia* is among the few plants that cope with the adverse conditions such as toxic metal in water. Previous study showed that *T*. *angustifolia* could endure a certain degree of toxic metal exposure with no visual toxic symptoms, and some kinds of toxic metals, i.e. Cd and lead, could increase plant height and biomass [21]. Our previous work also demonstrated that *T*. *angustifolia* was a metal-hyperaccumulator and showed high toxic metal tolerance. Therefore, *T*. *angustifolia* can be a preferred candidate to perform miRNA sequencing and analyze their role in toxic metal stress response. Cadmium is becoming a widespread and harmful toxic metal pollutant in recent years. However, there has been no report on systemic identification of Cd-responsive miRNAs and their targets in *T*. *angustifolia*, which may be because of the limited genome sequence data. The focus of this work is to analyze mRNAs, miRNAs as well as their targets involved in *T*. *angustifolia* Cd stress response. It will extend the current view on the molecular understanding of miRNA-guided regulation of plant toxic metal adaptation.

## Materials and Methods

### Plant materials, growth conditions and Cd exposure

Seeds of *T*. *angustifolia* were scattered in the pot and irrigated with tap water to keep humidity. After growing for 90 days, uniform seedlings were chosen and transferred to aerated nutrient solution (quarter-strength MS solution) at 20/15°C (day/night), with a light intensity of 200 μmol m^−2^s^−1^ and 12 h photoperiod for another 28 days. Uniform seedlings were then chosen and incubated in a nutrient solution containing 0 and 250 μM CdCl_2_ for different times. The concentrations of CdCl_2_ used in this study were determined in pilot experiments (S1 Fig). As shown in S1 Fig, exposure seedlings to different concentrations of CdCl_2_ could cause growth inhibition and resulted in significant increase in electrolyte leakage and thiobarbituric acid reactive substance (TBARS) content. As 500 and 750 μM CdCl_2_ caused serious plant wilt (S1A Fig), we chose 250 μM CdCl_2_, which induced nearly 50% increase in Electrolyte leakage and TBARS content, as the stress concentration (S1B–S1E Fig). For transcriptome and small RNA sequencing, the roots, stems and leaves of seedlings were separately harvested after 1, 6, 12 and 24 h of exposure and immediately frozen in liquid nitrogen for analysis.

### Small RNA and mRNA-seq library preparation and sequencing

Total RNA was extracted from frozen roots, stems and leaves of *T*. *angustifolia* with Trizol (Invitrogen, Carlsbad, CA, USA). Two sets of total RNA were prepared, with one derived from the original RNA pool prepared from Cd-treated tissues (roots, stems and leaves) (+Cd) at 1, 6, 12 and 24 h time points and the other from the RNA pool derived from Cd-free tissues (-Cd) at the same time points. For mRNA-seq, the two sets of total RNA were directly pooled together for cDNA library construction and sequencing at the Beijing Genomics Institute (BGI, Shenzhen, China), following the manufacture’s protocols. Briefly, beads coated with oligo (dT) were used to isolate poly (A) mRNA from the total RNA. A fragmentation buffer was added to interrupt the mRNA and thereby generate fragments in the size range 100–400 bp. The resulting fragments served as a template for the synthesis of the first strand cDNA, employing as primer random hexamers (N6). Second strand cDNA was synthesized using a SuperScript Double-Stranded cDNA Synthesis kit (Invitrogen, Camarillo, CA), after which it was purified using a QiaQuick PCR extraction kit (Qiagen, Hilden, Germany) and resolved with elution buffer for end reparation and poly (A) addition. The products were ligated with one another using sequencing adapters, and after agarose gel electrophoresis, a suitable size range of fragments were selected for PCR amplification. The resulting library was sequenced using an Illumina HiSeq 2000 device.

To generate the small RNA libraries, the samples were then subjected to 15% denaturing polyacrylamide gel electrophoresis, after which the sRNA fragments of 18–28 nt were isolated from the gel and purified. Next, the sRNA molecules were ligated to a 5’ adaptor and a 3’ adaptor sequentially and then converted to DNA by RT-PCR. Finally, 20 μg product of RT-PCR was sequenced directly using Solexa 1G Genome Analyzer according to the manufacturer’s protocols (BGI, Shenzhen, China). Sequence data from this article can be found in the GenBank data libraries under accession numbers GSE65091 (small RNA) and SRX847379 (transcriptome).

### mRNA analysis

S2A Fig summarizes the overall data analyses performed with the mRNA-seq libraries. Data filtering includes removing adaptors and low-quality reads from raw reads. Statistics analysis and evaluation of data, including total raw reads, total clean reads, Q20 percentage, N percentage and GC percentage. Transcriptome *de novo* assembly is carried out with short reads assembling program-Trinity. Briefly, image data output from the sequencing device was transformed into raw reads and stored in FASTQ format. These data were filtered to remove raw reads that included adapter sequence or were of low quality. The assembly of the transcriptome was achieved using the short-read assembly program Trinity [22]. The unigenes are divided into either clusters or singletons. BLASTX [23] alignment (applying an Evalue of less than 10^−5^) between each unigene sequence and those lodged in Nr (non-redundant protein database, NCBI), Nt (non-redundant nucleotide database, NCBI), Swiss-Prot, GO (gene ontology, http://www.geneontology.org/) and COG (clusters of orthologous groups) databases were performed, and the best alignments used to infer the unigene’s directionality. Where the outcome from the various databases conflicted with one another, the priority order applied was Nr, Swiss-Prot, COG. Where no alignment was possible, the software tool ESTScan [22,24] was used to assign directionality.

Functional annotation was assigned using the protein (Nr and Swiss-Prot), COG and GO databases. BLASTX was employed to identify related sequences in the protein databases based on an Evalue of less than 10^−5^. The COG database is an attempt to classify proteins from completely sequenced genomes on the basis of the orthology concept [25]. GO’s aim is to standardize the representation of genes and their products, by insisting on a controlled vocabulary and a strictly defined concept [26]. The annotations acquired from Nr were processed through the Blast2GO program [27] to obtain the relevant GO terms, and these were then analyzed by WEGO software [28] to assign a GO functional classification and to illustrate the distribution of gene functions.

### Small RNA analysis

S2B Fig summarizes the overall data analyses performed with the sRNA libraries. Clean reads were screened from raw sequencing reads by removing contaminated reads including sequences with 5’-primer contaminants, without the inserted tag, with poly(A) tails, either shorter than 15 nt or longer than 30 nt. After removing the adaptor/acceptor sequences, filtering the low quality tags and cleaning up the contamination formed by the adaptor-adaptor ligation, the occurrences of each unique sequence read were counted as sequence tags. The remaining unique RNAs were mapped to the *T*. *angustifolia* mRNA transcriptome sequences using SOAP 2.0 program [29]. Sequences with a perfect match were retained for further analysis. All these sequence tags were compared with the sequences of non-coding RNAs including ribosomal RNA (rRNA), transfer RNA (tRNA), small nuclear RNA (snRNA), small nucleolar RNA (snoRNA) which were available in Rfam (http://www.sanger.ac.uk/software/Rfam) [30] and the GenBank noncoding RNA database (http://blast.ncbi.nlm.nih.gov/) to classify degradation fragments of noncoding RNA. The rest of the sequences which could match *T*. *angustifolia* transcriptome sequencing data were searched for miRNA sequences using miRBase 21 (http://www.mirbase.org/index.shtml) [31]. The steps were summarized in S1 Table.

### Prediction of novel miRNAs

Prediction of *T*. *angustifolia* miRNAs was conducted using criteria that were previously developed for plant miRNA prediction [32]. MicroRNA precursors have characteristic fold-back structures that can be used to predict novel miRNAs. The prediction was implemented in the Mireap program developed by the BGI. Some key conditions were summarized in S1 Table. The expression of novel miRNA is produced by summing the count of those miRNAs with no more than 3 mismatches on the end of 5' and 3' and no mismatch in the middle from the alignment result.

### Prediction of miRNA targets

The identified known miRNAs and predicted novel miRNAs were used to interrogate sequences for target sites on the psRNAtarget web server (http://biocomp5.noble.org/psRNATarget/) using *T*. *angustifolia* transcript sequencing data. The target transcripts containing complementary sequences of miRNAs were determined with previously established criteria [33–35]. The criteria for target prediction were summarized in S1 Table. The functional category of obtained target sequences was annotated against the COG database (http://www.ncbi.nih.gov/COG/) using BLAST program with a cutoff of E value <1e^-5^.

### Differential expression of conserved miRNAs

To investigate the differentially expressed miRNAs between libraries, firstly, each identified miRNAs read count was normalized to the total number of miRNA reads in each given sample. Then, the Bayesian method was applied to infer the statistical significance value [36]. This approach was developed for analysis of digital gene expression profiles in previous studies and accounts for the sampling variability of tags with low counts. The procedures were summarized in S1 Table.

### Confirmation of mature miRNAs and target genes expression

For determination of miRNA expression, RNAs were reverse-transcribed by One Step PrimeScript miRNA cDNA Synthesis Kit (TaKaRa), which added a ploy (A) tail to the 3’-end of miRNA and with transcription leading by a known oligo-dT ligate. SYBR Premix Ex Tag II (TaKaRa) was used for Real-time quantitative RT-PCR (RT-qPCR). Small nuclear RNA U6 was used as an internal reference. For determination of target gene expression, 4 μg of total RNA was reverse-transcribed using an oligo(dT) primer and SuperScript Reverse Transcriptase (Invitrogen, USA). Real-time quantitative RT-PCR reactions were performed using a Mastercycler ep *realplex* real-time PCR system (Eppendorf, Hamburg, Germany) with SYBR *Premix Ex Taq* (TaKaRa Bio Inc., China) according to the manufacturer’s instructions. The relative expression level was presented as values relative to control samples, after normalization to *Actin* transcript levels. The primers were listed in S2 Table. Data are means ± SE from at least three independent experiments. Six biological replicates were analyzed in each set of experiments. The *t*-test (*P* < 0.05) was selected for statistical analysis.

## Results

### Transcriptome sequencing and annotation

In order to obtain global mRNAs from *T*. *angustifolia*, a normalized cDNA pool was constructed from total RNAs of seedlings treated with or without Cd. The cDNA was sequenced on an Illumina HiSeq 2000 platform. We obtained a total of 57,608,230 raw reads from sequencing (S3 Table). After removal of low quality regions, adaptors and all possible contaminations, more than 54 million clean reads (94.3% of the raw reads) was generated with a Q20 (base quality more than 20) percentage of 98.25 and a GC content of 47.47% from the transcriptome library. The total length of the read is more than 56 Mb. By *de novo* assembling, a total of 146,562 contigs were obtained with an average length of 385 nt (S3 Table). To join further sequences and remove any redundant sequences, contigs were connected to yield 102,473 unigenes with a total length of 94 Mb and average length of 919 nt. Length distributions of these unigenes and contigs are shown in S3 Fig Most unigenes (52.54%) are longer than 500 bp while 21.67% contigs are longer than 500 bp.

All assembled unigenes were then aligned by Blastx to the Nr database for annotation, as well as to the NT, Swiss-Prot, GO, KEGG and COG. Results indicated that a total of 60,303 unigenes are annotated, which represented about 58.85% of the total unigene set (S4 Table). Most of these unigenes are annotated against NR database. The *E* value distribution of the top hits in the Nr database showed that 32.6% of the mapped sequences had strong homology (<1e^-100^) (S4A Fig). Among annotated unigenes, 18.7% had a similarity higher than 80% (S4B Fig). For species distribution, more than 50% sequences have top matches with sequences from Japanese rice (15.7%), followed by Vitis vinifera (15.6%) (S4C Fig).

**Fig 1:**
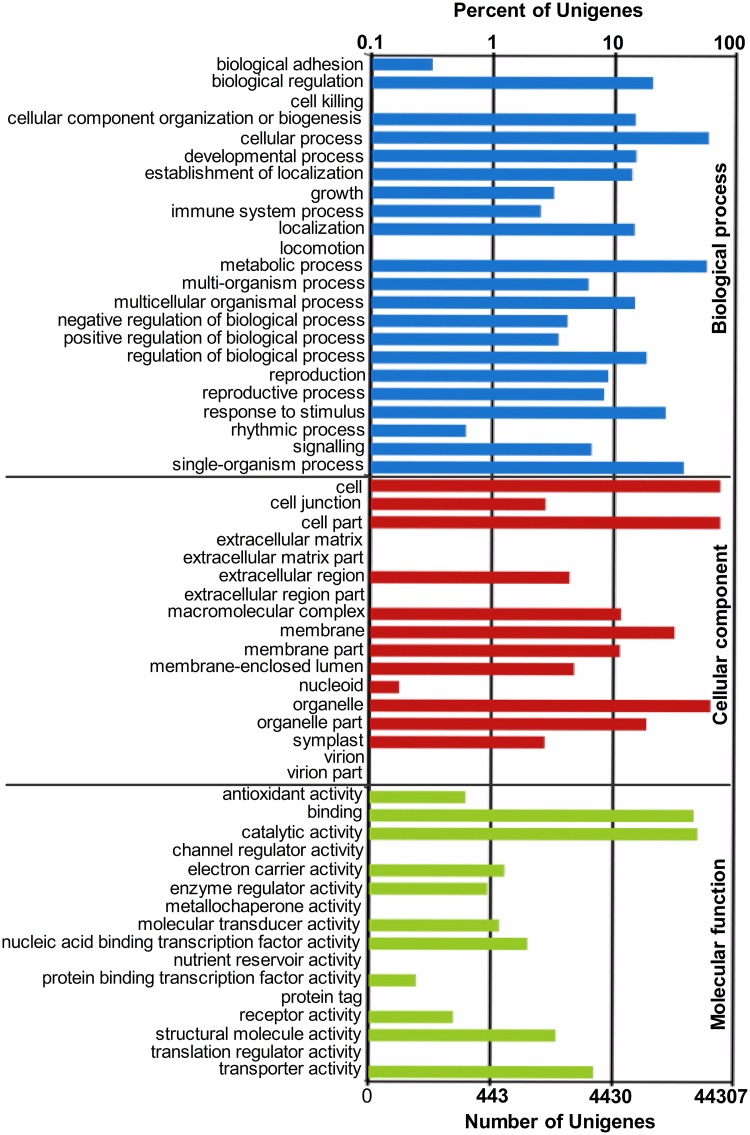
Histogram presentation of gene ontology (GO) classification. The up and down x-axis indicates the percentage and number of a specific category of genes in that main category respectively.

Based on Nr annotations, 60,303 unigenes were assigned with GO terms. All GO terms are allocated into three main GO categories including biological, cellular component and molecular function (Fig 1). Most of the identified transcripts appeared to be genes involved in biological processes. Cellular process and metabolic process were well-represented in biological processes category with percentage of 43.28 and 42.13%. Among functional groups of cellular component, GO terms are predominantly associated with cell, cell part and organelle. The molecular function category was dominated by DNA binding (34.03%) and catalytic activity (36.61%).

**Fig 2:**
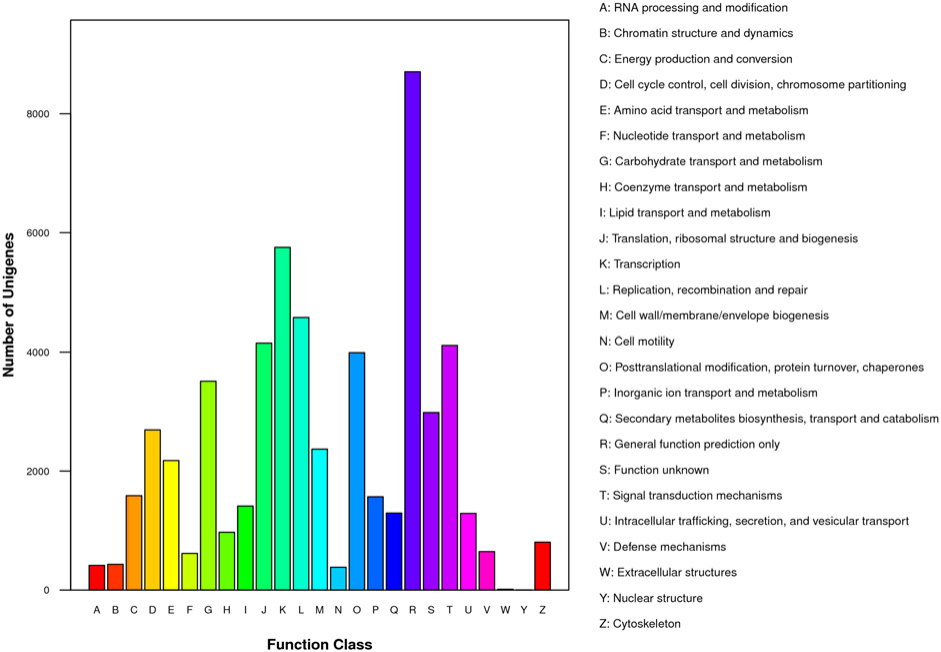
Cluster of Orthologous Groups of proteins (COG) function classification of unigenes in All-unigene. The horizontal coordinates are function classes of COG, and the vertical coordinates are numbers of unigenes in one class. The notation on the right is the full name of the functions in x-axis.

All the unigenes were also mapped to the COG database to further evaluate the effectiveness of the annotation process and understand gene function distribution characteristics of the species (Fig 2). These unigenes were classified into 25 COG categories. The generation function prediction only category represented the most common category. Extracellular structures and nuclear structure represent the smallest COG categories.

Kyoto Encyclopedia of Genes and Genomes (KEGG) analysis can help to further understand the biological functions based on genes associated with biochemical pathways. In this work, 38,058 unigenes were assigned to 128 KEGG pathways (S5 Table). Among them, metabolic pathway (25.56%) and biosynthesis of secondary metabolites (10.58%) were the largest two pathway groups. These results would provide valuable information to study the environmental stress response mechanisms in *T*. *angustifolia*.

### High-throughput sequencing of small RNAs

**Fig 3:**
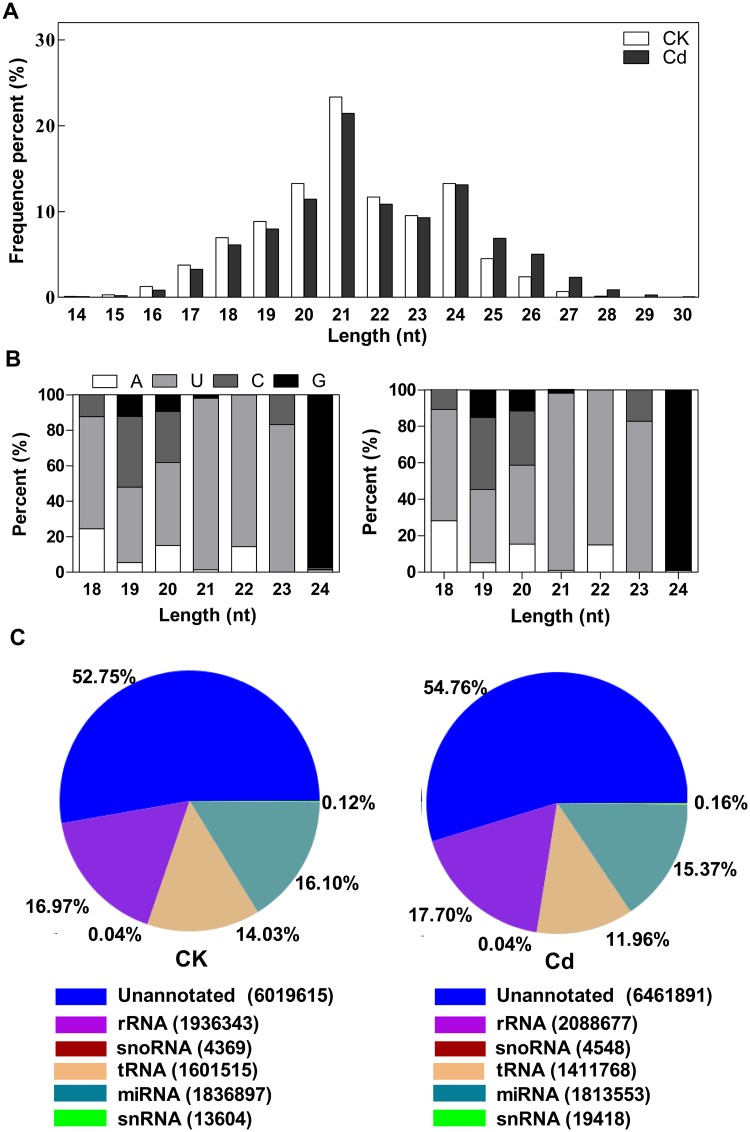
Length distribution (A), first nucleotide bias (B) and composition (C) of the small RNA in CK and Cd libraries. A, Average percentage (Y-axis) of redundant sequences of 14–30 nt length (X-axis) in CK and TR libraries. B, Base bias on the first position among small RNA with certain length. Each color in the figure shows the sRNA tags whose first base is a certain base. C, Summarization of all alignments. To make every unique small RNA mapped to only one annotation, we follow the following priority rule: rRNA etc (in which Genbank >Rfam) > known miRNA > repeat > exon > intron.

Two separate sRNA libraries were generated from *T*. *angustifolia* seedlings treated with (Cd) or without (CK) Cd-exposure and sequenced by Illumina sequencing technology. The sequencing acquired 12,128,241 reads from the CK library and 12,392,054 reads from the Cd library. After removing adaptor/acceptor sequences, filtering out low quality tags and cleaning up the contamination formed by the adaptor-adaptor ligation, 11,412,343 clean reads and 11,799,855 clean reads were obtained from CK library and Cd library respectively (S6 Table). In both libraries, the 21-nt and 24-nt classes showed the highest degree of redundancy (Fig 3A), suggesting that sRNAs in these size classes are often produced from precursors. The size distribution of all sRNAs was found to be uneven with a length range of 14–30, where the majority was 20–24 nt long. Analysis of the first nucleotide of 18–25 nt long sRNAs revealed that many sRNAs started with uridine (U) at their 5’-ends (Fig 3B). We further screened the clean data against rRNAs, snoRNAs, snRNAs and tRNAs in the NCBI Genebank database and Rfam database (Fig 3C; S6 Table) resulting in 7,856,512 (CK) and 8,275,444 (Cd) reads remaining for further analyses. Among the remaining sequences, 1,836,897 sequences of CK library and 1,813,553 sequences of Cd library were similar to known miRNAs from other plant species that had previously been deposited in miRBase 21.

### Conserved miRNAs and evolutionary conservation

Because the cleavage effect of RNase III-like nuclease on miRNA precursors was imprecise, the above candidate miRNA sequence probably came from the same precursor. We thus further clustered those sequences based on sequence similarity. We determined that the sequence with the dominant number of reads in a cluster was likely to be the real sequence and the expression of miRNA was generated by summing the count of tags which could align to the temporary miRNA database within two mismatches. These bioinformatics analyses resulted in 114 conserved miRNAs belonging to 99 families in both libraries (S7 Table). Most miRNA families had only one member. The frequency of diverse members in same or different miRNA families varied drastically. The expression levels of a few miRNAs, such as miR156a and miR166a, were extraordinarily high in both libraries. However, some miRNAs, such as miR858 and miR1870 had only less than 10 reads.

To explore the evolutionary features of the identified known miRNAs, we performed extensive comparisons against published miRNAs from other species (S8 Table). At present, there is no available miRNA data of *T*. *angustifolia* deposited in the public miRNA database. Comparison results showed that ten miRNAs were highly evolutionarily conserved in plant from different divisions, with miR171 being the dominant family detected from 30 species, followed by miR156, miR160, miR159, miR166, miR167, miR408, miR319, miR396, and miR390. This suggested that these miRNAs could perform important and conserved effect in diverse species. About one fifth (21 out of 99) miRNA families showed a lack of conservation in miRNAs evolution when comparing to 29 other plant species. Interestingly, *T*. *angustifolia* and other selected monocotyledons shared 45 evolutionarily conserved miRNAs, and 18 of which had no ortholog in other analyzed plants, indicating that these 18 miRNAs were probably involved in regulation of monocotyledons-specific processes.

### Identification of pre-miRNAs and *T*. *angustifolia*-specific miRNA families

To identify putative pre-miRNA sequences in *T*. *angustifolia*, the sRNA library was matched against above assembled sequence contigs derived from our transcriptome sequence data. The secondary structures of potential miRNA precursors were obtained using m-fold [37]. There were 150 potential pre-miRNAs that met the requirements for miRNAs (S9 Table). The minimal folding free energy (MFE) of these predicted pre-miRNAs ranged from -28.2 to -127 kcal/mol with an average of -55.98 kcal/mol. The putative pre-miRNA sequences were varied mainly in length from 68 to 255 nt (S9 Table). For 150 miRNA duplex-like pairs we predicted, 50 were identified as known full-length plant pre-miRNA sequences along with 26 miRNAs anchored in the 3p-arm and 37 miRNAs in the 5p-arm (S9 Table).

**Fig 4:**
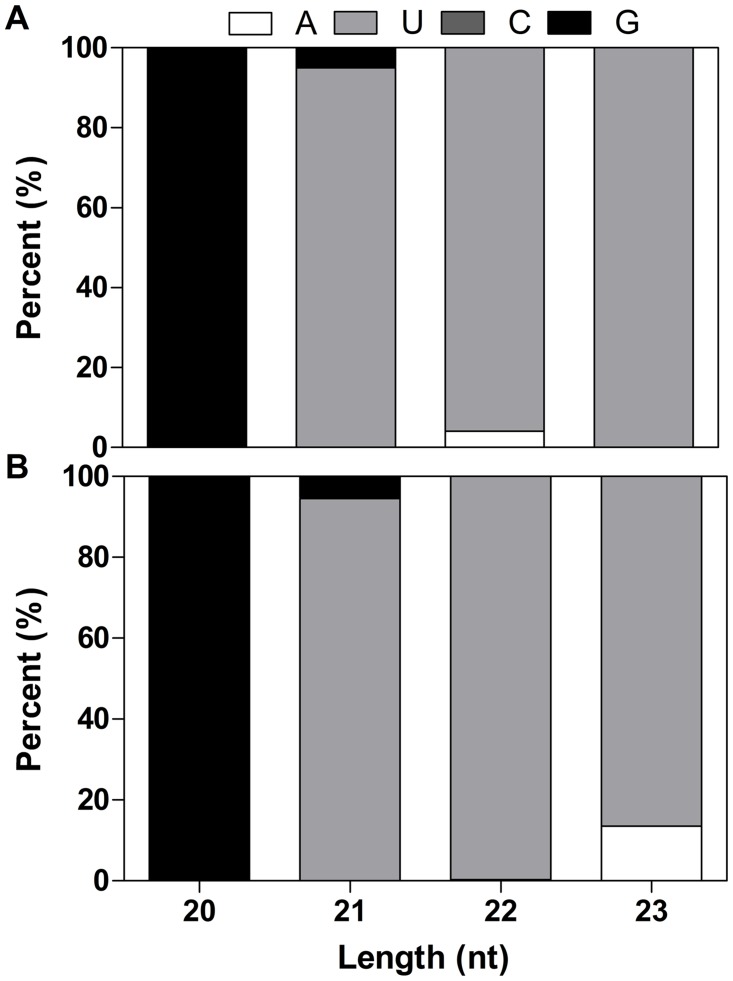
First nucleotide bias of novel miRNA in CK (A) and Cd (B) libraries. Base bias on the first position among novel miRNAs with certain length. Each color in the figure shows the percentage of miRNAs whose first base is a certain base.

In addition to conserved miRNAs, we identified 66 potential pre-miRNAs that met the requirements for new miRNAs (S9 Table). These results revealed the existence of 41 novel miRNAs belonging to 31 miRNA families in *T*. *angustifolia* (S9 Table). We identified 9 pairs of miRNA candidates that come from 5p-arm and 3p-arm of the pre-miRNA respectively. The lengths of these 41 novel miRNAs ranged from 19 to 23 nt with 67.5% being 21 nt in length, a classical length of miRNA. Moreover, the first nucleotide bias analysis showed that uracil was the most frequently used first nucleotide in novel miRNAs, which is also a hallmark of miRNA (Fig 4) [38]. All of novel miRNAs have more than one miRNA precursors. For example, novel_mir_20 have two precursors. The most abundant novel miRNA yielded 28,015 reads in CK library, suggesting an important role in *T*. *angustifolia*.

### Identification of IsomiRNAs

Variants of miRNAs, called isomiRNAs, were considered to be a consequence of inaccuracies in Dicer pre-miRNA processing, and were proved to have different functions of their previously reported miRNAs due to differential associations with argonaute (AGO) proteins. In this study, the sequencing results also revealed that the majority identified miRNAs showed length and sequence heterogeneity. The number and abundance of each isomiRNA corresponding to conserved and novel miRNA are listed in S10 and S11 Tables, respectively. The results showed that miRNAs differ significantly from each other in the number and abundance of isomiRNAs. Among them, the known pre-miRNA of miRNA166a and the novel pre-miRNA of novel_mir_8 produced more isomiRNAs than the other pre-miRNAs. In the majority of the identified miRNAs, frequent nucleotide variations were observed particularly in the 5’ end, mainly in the form of missing nucleotides and/or terminal additions of nucleotides. It is interesting to note that in some miRNA families, the most abundant miRNA was not the canonical miRNA described for other species.

### Target prediction of miRNAs

As miRNA target prediction is critical for gaining insight into the regulatory functions of miRNAs, in this study, we searched unigene sequences derived from high-throughput sequencing of *T*. *angustifolia* for the putative target genes of its miRNAs. Based on perfect or near perfect complementarity between miRNAs and their targets, a total of 764 unigenes was predicted as potential targets of 89 known plant miRNAs and 21 novel miRNAs, with an average of 6.95 targets per miRNA (S12 and S13 Tables). The MFEs for the miRNA:mRNA hybrids ranged from -48.8 to -21.3 kcal/mol.

**Fig 5:**
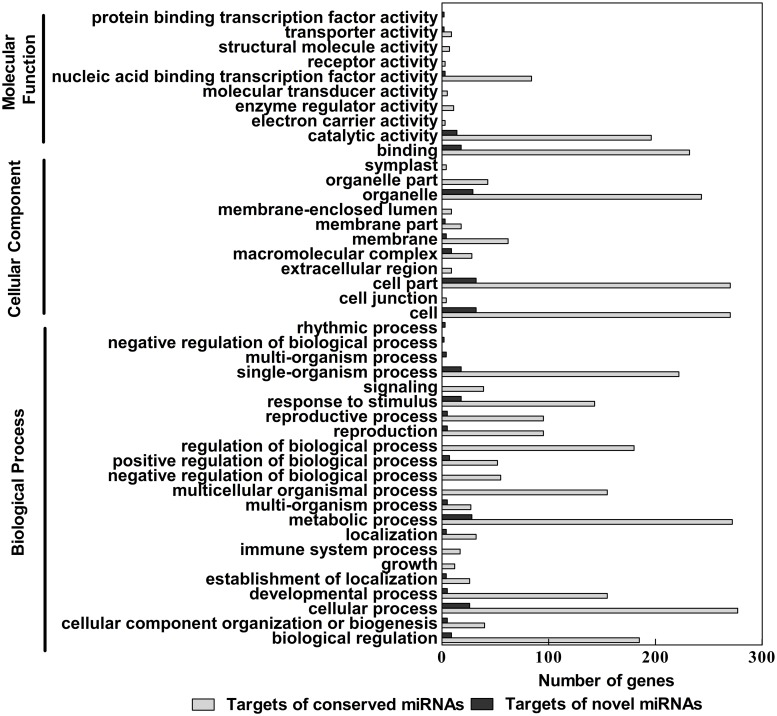
Targets of the miRNAs identified in *T*. *angustifolia*. The number of genes for each Gene Ontology (GO) term is relative to the total number of contigs from each gene category.

For comprehensive annotation, all of the identified targets were analyzed by using BLASTX against the protein database, followed by a GO analysis to evaluate their putative functions (Fig 5). We found that all these target genes were involved in 43 different molecular functions belonging to biological processes, cellular components and molecular function. GO analysis results demonstrated that 161 target genes could be involved in stimulus response processes. A significant number of the predicted targets were poorly characterized genes, suggesting possible new roles for these miRNAs in *T*. *angustifolia*.

### Different expression profiles of small RNAs in CK library and Cd library

**Fig 6:**
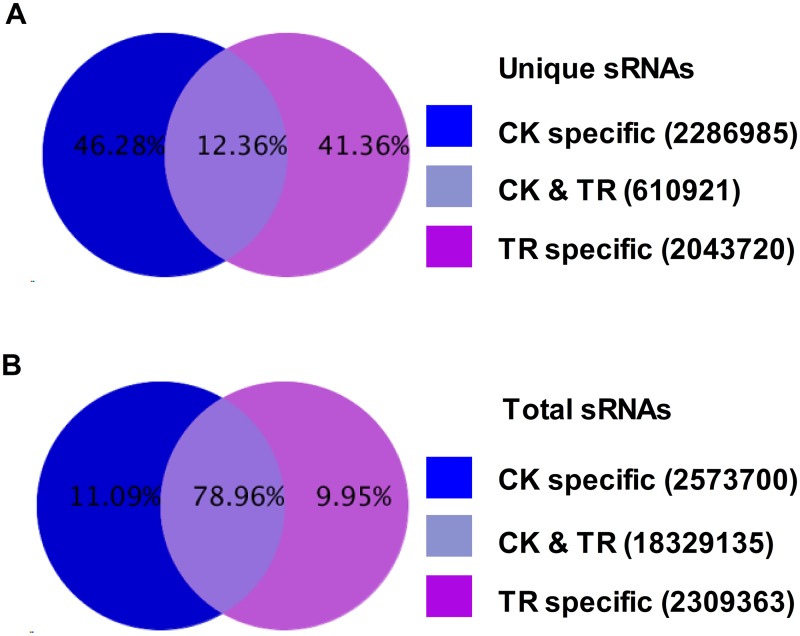
Common and specific sequences between CK and Cd library. Summarise the common and specific tags of two libraries, including the summary of unique tags and total tags.

To identify differently regulated sRNAs under Cd stress in *T*. *angustifolia*, we summarized the common and specific sequences between two libraries. CK and Cd library shared 18,329,135 (78.96%) sequences among the total sRNAs, which indicated that the majority of small RNAs were shared (Fig 6). However, the shared unique sRNAs were much smaller, only occupying 12.36% of the total unique sRNAs. In these unique sRNAs, the count of CK-specific sRNA was 2,286,986 reads (46.28%), which is higher than Cd-specific sRNAs 2,043,720 reads (41.36%). This means that Cd stress induces some small RNAs and represses other miRNAs, and that more unique small RNAs are repressed than induced. The size distribution of small RNAs in two libraries was slightly different (Fig 3). The contents of two most abundant small RNA classes, 21-nt and 24-nt, in CK library were increased compared to those in the Cd library.

**Fig 7:**
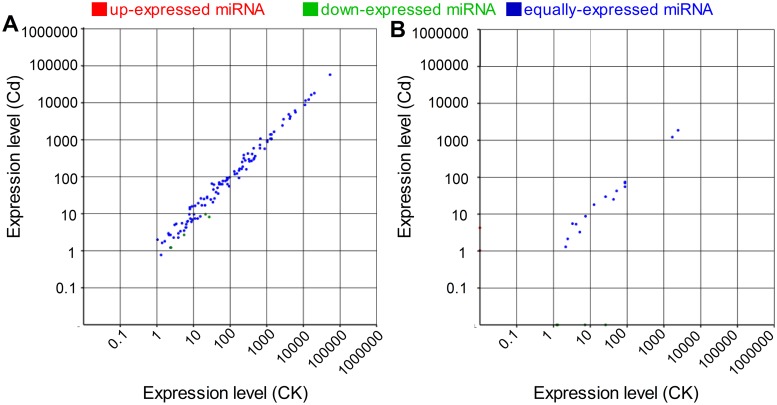
Scatter-plot graphs represent the conserved miRNA (A) and novel miRNA (B) differential expression patterns between control (CK) and cadmium (Cd) stress. The X axis indicates normalized gene expression levels in control and the Y axis indicates the normalized gene expression levels (per transcript) in Cd-stresses tissues. The dots which are located at the upper and lower side of the diagonal line reflects the changes in the expression levels of miRNA genes; above the diagonal line, indicating up-regulation whereas below the diagonal line indicating down-regulation. For miRNA deep-sequencing experiment, the fold change cut-off was set at 1.5.

**Fig 8:**
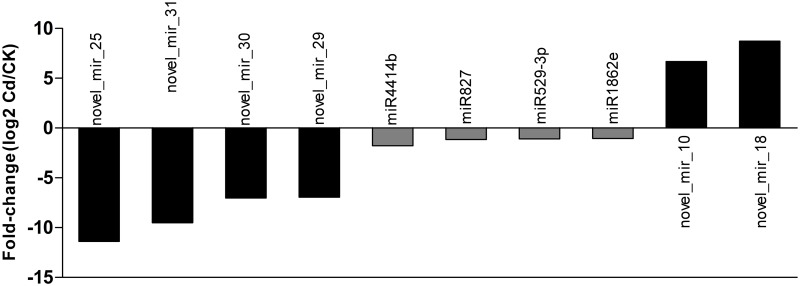
The differentially expression of significant changed miRNAs between CK and Cd libraries. The expression of miRNAs in two libraries were first normalized to get the expression of transcript per million (TPM). Then fold change was calculated according to the formula: Fold change = log2 (treatment/control).

We then compared the accumulation level of miRNAs between the two libraries. The expression of miRNAs in two libraries was shown by plotting Log2-ratio figure (Fig 7) and detailed in S14 Table. Statistical analyses demonstrated that 4 of 99 conserved miRNAs and 6 of 41 novel miRNA were expressed significantly different between the two libraries (Fig 8). Among them, eight miRNAs including novel_mir_25-3p, novel_mir_31-3p, novel_mir_30-5p, novel_mir_29-5p, miR4414b, miR827, miR529-3p and miR1862e were down regulated, whereas the other two miRNAs (novel_mir_10-5p and novel_mir_18-3p) were up-regulated by Cd stress. 4 miRNAs were only expressed in the Cd library, while 2 miRNAs were specifically expressed in CK library, suggesting that these miRNAs might be induced or repressed under Cd stress. We also noticed that all the miRNAs with the greatest change (>5 fold) in expression levels are novel miRNAs.

### Validation and expression patterns of miRNAs and their target genes

To confirm the accuracy and reliability of sRNA-seq results, 10 differentially expressed miRNAs were analyzed by RT-qPCR (S15 Table). The expression profiles of those miRNAs matched the sRNA-seq data closely.

We also examined the expression patterns of corresponding target genes of these miRNAs. As shown in S15 Table, miRNA-mediated regulation of target gene expression level appears to be occurring, suggesting that the data generated from RNA-Seq assay of this study are reliable to be used to investigate Cd-induced miRNA changes in *T*. *angustifolia*.

## Discussion

### Transcriptome analysis of *T*. *angustifolia*

At present, genome data of *T*. *angustifolia* are unavailable, and other sequence data, i.e. EST data, are also rarely found in the public database, which largely limited the study in *T*. *angustifolia* including sRNA research. Illumina sequencing is a powerful tool for gene discovery, which could provide enormous transcript sequence information. For more thoroughly investigated sRNA in *T*. *angustifolia*, we deep sequenced a normalized cDNA pool constructed from mixed *T*. *angustifolia* seedlings treated with or without Cd. With this technology, more than 57 million raw data were produced, from which a total of 102,473 unigenes was annotated by alignment analysis to the NT, Swiss-Prot, GO, COG and KEGG (S3 and S4 Tables; S3 and S4 Figs). Among them, 52.54% unigenes and 21.67% contigs are longer than 500 bp. According to the result of GO and COG classification (Figs 1 and 2), these annotated unigenes participated in various biological process. The results of KEGG also revealed the various genetic processing pathway information and environmental information processing (S5 Table). Functional classification and pathway enrichment can contribute to a better understanding of gene function in the network of gene interactions. These unigene and contig sequences would provide more valuable gene information and supplement the transcriptome sequence for pre-miRNA identification. This is also the first exploration to gain insight into the transcriptome of *T*. *angustifolia*.

### The conserved and novel miRNAs in *T*. *angustifolia*

MiRNAs are a class of short non-coding, endogenous RNAs that play key roles in many biological processes. Although miRNAs have been studied extensively in the past several years, the miRNAs in *T*. *angustifolia* have been poorly understood. In this study, two small RNA libraries were constructed from *T*. *angustifolia* with or without Cd exposure and sequenced using high-throughput Solexa technology. As no small RNA sequence in *T*. *angustifolia* was reported previously, we first thoroughly summarized and analyzed the small RNAs in combined sequencing data.

Solexa sequencing resulted in a total of 12,128,241 and 12,392,054 high quality reads from CK and Cd library respectively. Most of the sequences from the two libraries are between 20–24 nt (Fig 3). Similar to previous reports [39–42], most of the miRNAs were 21 and 24 nt in size and started with uridine (U) at their 5’-ends, which is one of the important characteristic features of miRNAs. However, in contrast to previous reports that the 24-nt sRNAs are more abundant in plants than the 21-nt class [43], both libraries showed a unexpected peak at 21 nt. This difference might be attributed to the different sRNA biogenesis pathways in various plants [44].

To identity the known miRNAs in *T*. *angustifolia*, we compared the data from the two libraries to known miRNAs in miRBase 21.0 (http://www.mirbase.org/). Our data revealed the existence of 114 conserved miRNAs belonging to 99 families in both libraries (S7 Table). The frequencies of miRNA varied from 7 reads (miR1870-5p) to 229669 reads (miR156a), indicating that expression varies significantly among different miRNAs. Besides, different members in same family also showed clearly different, probably because the expression is tissue- and/or developmental-stage specific. In comparison with other plant species, some abundant miRNAs, for example miR156, miR166 and miR168 in *Avicennia marina*, peanut and *Brachypodium*, also frequently appeared in our dataset [44–46]. By extensive comparisons between different plants species, we found that ten conserved miRNAs were highly evolutionarily conserved in plants from different divisions. Moreover, we also identified 18 monocotyledons-specific miRNAs and 21 *T*. *angustifolia specific miRNAs*. Well-evolutionarily conserved miRNAs often retain homologous target interactions and perform analogous molecular functions in the long process of evolution [47]. As shown in previous studies [48, 49], these evolutionarily conserved miRNAs mainly regulated a series of target genes that is greatly associated with the basic functions for normal growth and development of plant, and could be mobilized towards adaptive responses to stress. Based on the functions of these evolutionarily conserved miRNAs known in other plants, we can infer the functions of miRNAs in *T*. *Angustifolia*, due to these miRNAs had been reported to remain constant during plants diversification [47]. This will also provide an opportunity to inspect the evolution of these miRNA families during the divergence of plants.

Since details of the *T*. *angustifolia* genome sequence remain limited, the produced transcriptome of *T*. *angustifolia* were used as a sequence reference to identify putative pre-miRNA sequences. The candidate pre-miRNAs were predicted by exploring the secondary structure, MFE and minimal folding free energy index (MFEI) using Mireap software (https://sourceforge.net/projects/mireap/). In all, 150 potential pre-miRNAs were identified, with an average MFE of -55.98 kcal/mol, which is similar to the free energy values of other plant miRNA precursor and are apparently lower than other types of RNAs such as tRNAs, rRNAs and mRNAs [50]. Among the 150 miRNA duplex-like pairs, 50 were identified as the pre-miRNA of conserved miRNA (S9 Table). 26 miRNAs were anchored in the 3p-arm and 37 miRNAs in the 5p-arm of these pre-miRNAs. Due to the limitation of transcriptome data, pre-miRNA of 64 conserved miRNAs can not be found. In addition to conserved miRNAs, 66 of these potential pre-miRNAs were characterized as novel pre-miRNA in *T*. *angustifolia*. As a result, 41 potential novel miRNAs belonging to 31 miRNA families were identified in *T*. *angustifolia* (S9 Table). For 9 pairs of this candidate miRNAs, we found both miRNAs come from 5p-arm and 3p-arm of the pre-miRNA, which adding weight to the authenticity of the predicted miRNA candidates. Consistent with previous reports that species-specific miRNA are usually expressed at a low level, the most abundant novel miRNA only yielded 28,015 reads in CK library [51]. The low abundance of these novel miRNAs might suggest that they play specific roles in specific tissues or developmental stages.

MiRNAs were initially thought to have a specific sequence of a defined length. However, with the discovery of miRNAs from different species, it was shown that Dicer mediated pre-miRNA processing was inaccuracy, which could result in miRNA isoforms with additional nucleotides in the 5’ or 3’ terminus from the same locus [52,53]. This might provide the chance for some miRNAs to base pair with other target mRNAs, exhibiting a species-specific regulatory pattern [54]. In the present study, by aligning the sRNA library with identified *T*. *angustifolia* pre-miRNAs, we also estimated the number and abundance of each isomiRNA corresponding to conserved miRNAs (S10 and S11 Tables). We found that in some cases, the most abundant sequences among all unique sequences mapped to the identified pre-miRNAs of *T*. *angustifolia* were not annotated as miRNA sequences in miRBase (S10 and S11 Tables). This suggests that these isoforms might involve in divergent functions and might be necessary at different levels according to the species, timing, tissue and/or other situations.

To clarify the biological functions of the conserved as well as the novel miRNAs identified in *T*. *angustifolia*, this study presented the first transcriptome-based analysis of miRNA targets in *T*. *angustifolia* (S12 and S13 Tables). Consistent with previous studies, many identified miRNA targets were transcription factors, for example MADS-box, MYBs and AP2-like factors, which were also identified as target gene of miRNAs (eg. miR159, miR164, miR169 and miR172) in other plant species [55, 56]. GO annotation showed that these putative target genes appeared to be involved in a broad range of biological processes. As expected, many conserved miRNAs targeted genes that involved in essential biological processes, such as cellular response to nitrate, maintenance of organ identity and so on. Unlike conserved miRNAs, the targets of novel miRNAs were enriched in some process related to stress response, such as response to redox state, DNA repair and cellular response to stress. Besides, we predicted many genes with unknown function. Careful analysis of these potential targets will contribute to our understanding of the role of miRNAs in *T*. *angustifolia*.

### The cadmium-responsive miRNA and their targets in *T*. *angustifolia*

Cadmium is becoming a widespread toxic metal pollutant that is harmful to all living organisms including plants and animals. To deal with this problem, phytoremediation using some high toxic metal tolerant plant, such as *T*. *angustifolia*, was proved to be a cheap and suitable method. To enhancing the effect of phytoremediation, the elucidation of the regulatory mechanisms underlying Cd response of these plants is becoming an urgent goal. MiRNAs have recently emerged as important modulators of plant adaptive response to toxic metal stress [57]. Understanding expression patterns of miRNAs in plant is necessary to discern miRNA-mediated regulatory pathways. In this work, a transcriptome-based analysis of miRNAs and corresponding targets in *T*. *angustifolia* seedlings under Cd exposure will facilitate our understanding of the regulatory mechanisms of *T*. *angustifolia* in response to Cd stress, which would provide useful information for improving the toxic mental resistance of *T*. *angustifolia* and other economically important plants.

Comparison of sequencing data of the two libraries showed that the distribution of different size sRNAs was different. CK library had a higher concentration in 21-nt and 24-nt sRNAs, which were most abundant small RNA classes in both lirbaries. Similar changes were also shown in a recent work which analyzed Cd-responsive miRNAs in radish [55]. This suggested that biogenesis of 21-nt and 24-nt sRNAs was sensitive to Cd stress. In contrast to 21-nt miRNAs which directly target mRNAs for cleavage, experimental data showed that a 24-nt miRNA could act to direct DNA methylation at their target genes. The distribution of kinds of sRNA classes exhibited the regulatory underlying of epigenetic adjustment. The changes of 21-nt and 24-nt sRNAs between the two libraries provided an explicit evidence to support the possibility that cross-interaction of multiple RNA-silencing pathways are involved in Cd-defense response.

As increasing evidence revealed that different members in a same family might involve in divergent functions and might be expressed at dramatically different levels, we did not pool together miRNAs of the same family, and analyzed them separately. The identified miRNAs between the two libraries displayed varied abundance from one another (Figs 7 and 8; S14 Table). For conserved miRNAs, only four miRNAs were significantly down-regulated by Cd stress with less than 1.7 fold changes. However, these conserved miRNAs usually had high levels of expression, even in the presence of Cd. Thus, slight fold changes might mean enormous gene regulation. In contrast with this, 4 novel miRNAs were negatively and 2 were positively regulated by Cd stress with over 5 fold changes. These novel miRNAs are expected to respond specifically to Cd stress in *T*. *angustifolia*. Intriguingly, 8 of 10 significantly changed miRNAs were down-regulated by Cd exposure. This observation suggests that *T*. *angustifolia* might enhance special pathway leading to the tolerance to toxic metal stress which usually under tight control of these miRNA for saving of energy.

Target prediction of the differentially expressed miRNAs could provide information on the biological processes regulated miRNA. The present study identified 12 transcript targets for 8 significantly changed miRNAs (S12 and S13 Tables). Two miRNAs including miR1862e and novel_mir_29 have no predicted targets. Besides the cause of limited transcriptome data, we can not rule out that they may silence their target activity via translational repression [58,59]. Some of these targets are shown regulating plant resistance to environmental stress. For example, miRNA827 targets a heat shock factor protein HSF8 which could enhance plant tolerance to biotic and abiotic stresses. However, most target genes of these Cd-responsive miRNAs were novel and had unknown function. We concluded that these Cd-responsive novel miRNAs and conserved miRNAs might participate in *T*. *angustifolia* specific processes which associated with its high toxic metal tolerance. Further studying of these target genes will facilitate our understanding of related mechanisms.

In conclusion, this is the first report on the transcriptome-wide identification of Cd-responsive miRNAs and their targets using transcriptome sequencing and sRNA sequencing in *T*. *angustifolia*. A total of 4 conserved miRNAs and 6 novel miRNAs were identified to be responsive to Cd stress. Ten transcript targets were predicted for 8 significantly changed miRNAs. Expression validation showed that miRNA-mediated regulation of target gene expression level appears to be occurring. Some target transcripts were functionally predicted to code biotic and abiotic stress-responsive protein. Meanwhile, most target genes of these Cd-responsive miRNAs were novel and had unknown function. These findings provided new information for further characterization of Cd-responsive miRNAs in *T*. *angustifolia*.

## Supporting Information

S1 Fig Dose-dependent effects of CdCl_2_ on the *Typha angustifolia* seedlings growth (A), electrolyte leakage (B, D) and thiobarbituric acid reactive substance (TBARS) contents (C, E). Seedlings were grown in nutrient solution in the presence of 0, 100, 250, 500, and 750 μM CdCl_2_ for 6 d. Photos showing response of *Typha angustifolia* seedlings to CdCl_2_ exposure were taken. Electrolyte leakage (B) and TBARS content (C) in roots were then analyzed. Time dependent changes of Electrolyte leakage (D) and TBARS content (E) under 250 μM CdCl_2_ exposure was also analyzed. Data are the means ± SE from three independent experiments. Six biological replicates were analyzed in each set of experiments. Within each set of experiments, bars with different letters are significantly different at P < 0.05, according to Duncan’s multiple range test.
(DOC)

S2 Fig Flow chart of the methodology adopted to transcriptome (A) and small RNA (B) analysis in *Typha angustifolia*. For transcriptome analysis, data filtering included removing adaptors and low-quality reads from raw reads. Transcriptome *de novo* assembly was carried out with short reads assembling program Trinity. Unigenes were annotated with the databases of non-redundant protein (Nr), NCBI non-redundant nucleotide sequence (Nt), Swiss-Prot, Kyoto Encyclopedia of Genes and Genomes (KEGG), Cluster of Orthologous Groups of proteins (COG) and Gene Ontology (GO). For small RNA analysis, the 49 nt sequence tags from sequencing were gone through the data cleaning analysis first, which includeed getting rid of the low quality tags, 5’ adaptor contaminants from the 50 nt tags, to get credible clean tags. The standard analysis annotated the clean tags into different categories and taken those which could not be annotated to any category to predict the novel miRNA and seed edit of potential known miRNA. After getting miRNA result, target prediction for miRNAs and GO enrichment and KEGG pathway for target genes were analyzed.
(DOC)

S3 Fig Length distributions of *Typha angustifolia* unigenes (A) and contigs (B). A, the horizontal coordinates are unigene or contig lengths and the vertical coordinates are numbers of unigenes. B, the horizontal coordinates are contig lengths and the vertical coordinates are numbers of contigs.
(DOC)

S4 Fig The E-value distribution (A), similarity distribution (B) and the species distribution (C) of NR annotation. Unigene sequences were firstly aligned to protein database NR by blastn, retrieving proteins with highest sequence similarity to the annotated protein in the database. Then counted and summarized annotation results.
(DOC)

S1 Table Criteria for prediction of conserved miRNAs, novel miRNAs, miRNA targets and analysis of expression level of miRNAs. (DOC)

S2 Table The sequences of primers for real-time RT-PCR. The primers of target genes were designed according to the respective sequence of contigs or Unigenes in transcriptome. The primers of miRNAs were designed according to the protocol of One Step PrimeScript miRNA cDNA Synthesis Kit (TaKaRa).
(DOC)

S3 Table Summary of Illumina transcriptome assembly for *T*. *angustifolia*. Q20, base quality more than 20. N50 of contigs or unigenes were calculated by ordering all sequences, then adding the lengths from longest to shortest until the summed length exceeded 50% of the total length of all sequences.
(DOC)

S4 Table Summary of functional annotation of the *T*. *angustifolia* transcriptome. The unigenes were annotated by aligning with the deposited ones in diverse protein databases including National Center for Biotechnology Information (NCBI) non-redundant protein (Nr) database, NCBI non-redundant nucleotide sequence (Nt) database, UniProt/Swiss-Prot, Kyoto Encyclopedia of Genes and Genomes (KEGG), Cluster of Orthologous Groups of proteins (COG) and Gene Ontology (GO). The overall functional annotation was summarized.
(DOC)

S5 Table KEGG pathway enrichment analysis in *T*. *angustifolia* transcriptome. Pathway ID, KEGG Pathway ID. KEGG pathway is divided two levels, level 1 owns major division of gene function, level 2 owns further division of gene functions within level 1.
(XLS)

S6 Table Statistics of small RNA sequences from CK and Cd libraries of *T*. *angustifolia*. Statistics of small RNAs from CK and Cd-infected sample in both total and unique reads. rRNA, ribosome RNA. tRNA, transporter RNA. snRNA, small nuclear RNA. snoRNA, small nucleolar RNA.
(DOC)

S7 Table Conserved miRNAs in *T*. *angustifolia*. Identified *T*. *angustifolia* conserved miRNAs and their reads in CK and Cd libraries. The identification was performed by selecting homologies to temporary miRNA database of *T*. *angustifolia* within two mismatches.
(DOC)

S8 Table Conserved miRNA families in *T*. *angustifolia* and across-species. Evolutionary conservation of 99 miRNA families identified in *T*. *angustifolia* within plant species reported in miRBase 21. Closely related species are shown in the same color. Colored boxes denote the presence of the indicated miRNA family.
(XLS)

S9 Table Predicted pre-miRNA of conserved and novel miRNAs in *T*. *angustifolia*. Location, location of miRNAs in the predicted pre-miRNA sequence. MFE, minimal folding free energy. Reference miRNA name (5p), sequenced miRNAs presented in 5’ arm of the hairpin pre-miRNA. Reference miRNA name (3p), sequenced miRNA presented in 3’ arm of the hairpin pre-miRNA. Seq, sequence of pre-miRNA. Seq (5p), sequence of reference miRNA presented in 5’ arm of the hairpin pre-miRNA. Seq (3p), sequence of reference miRNA presented in 3’ arm of the hairpin pre-miRNA.
(XLS)

S10 Table Conserved miRNA isoforms in CK and Cd libraries. Detected diverse isoforms of *T*. *angustifolia* conserved miRNAs. miRNA precursor information, in order: miRNA name, location and length of hairpin structure, MFE (minimal folding free energy), miRNA precursor sequence, stem loop structure name.
(XLS)

S11 Table Novel miRNA isoforms in CK and Cd libraries. Detected diverse isoforms of *T*. *angustifolia* novel miRNAs. miRNA precursor information, in order: miRNA name, location and length of hairpin structure, MFE (minimal folding free energy), miRNA precursor sequence, stem loop structure name.
(XLS)

S12 Table Predicted targets of conserved miRNAs in *T*. *angustifolia*. Potential targets of conserved miRNAs predicted using psRNATarget based on transcriptome data of *T*. *angustifolia*. Unigene or contig ID are used for target identification. Calculated MEFs (kcal/mol) are shown. MiRNA sequence in 5’-3’ sense were used to present miRNA:target pairing. Crosses (x) denote one-nucleotie mismatch. G:U base pairing is not considered a mismatch (o).
(XLS)

S13 Table Predicted targets of novel miRNAs in *T*. *angustifolia*. Potential targets of novel miRNAs predicted using psRNATarget based on transcriptome data of *T*. *angustifolia*. Unigene or contig ID are used for target identification. Calculated MEFs (kcal/mol) are shown. MiRNA sequence in 5’-3’ sense were used to present miRNA:target pairing. Crosses (x) denote one-nucleotie mismatch. G:U base pairing is not considered a mismatch (o).
(XLS)

S14 Table Differential expression of identified known and novel miRNAs between libraries. Different members of miRNA family with differential expressed pattern during *T*. *angustifolia* response to Cd. “sig-lable” means values are significantly different between two samples at *P* < 0.05 (one asterisks) or *P* < 0.01 (two asterisks).
(XLS)

S15 Table Relative expression pattern of selected conserved and novel miRNAs and their target genes. Samples for small RNA sequencing were used in RT-qPCR analysis. *U6* and *Actin* were used as internal reference genes for miRNA and target gene respectively. The expression levels of gene expression are presented as values relative to CK. Values are means of three independent experiments. Six biological replicates were analyzed in each set of experiments.
(XLS)
