# Supplementary material for: Transcriptome-Wide Identification of miRNAs and Their Targets from Typha angustifolia by RNA-Seq and Their Response to Cadmium Stress
Source: PLoS One. 2015 Apr 29;10(4):e0125462. doi: 10.1371/journal.pone.0125462 (PMC4414455; doi:10.1371/journal.pone.0125462)
Supplement: S1 Fig — Seedlings were grown in nutrient solution in the presence of 0, 100, 250, 500, and 750 μM CdCl2 for 6 d. Photos showing response of Typha angustifolia seedlings to CdCl2 exposure were taken. Electrolyte leakage (B) and TBARS content (C) in roots were then analyzed. Time dependent changes of Electrolyte leakage (D) and TBARS content (E) under 250 μM CdCl2 exposure was also analyzed. Data are the means ± SE from three independent experiments. Six biological replicates were analyzed in each set of experiments. Within each set of experiments, bars with different letters are significantly different at P < 0.05, according to Duncan’s multiple range test. (DOC) [file pone.0125462.s001.doc]

**Figure S1**


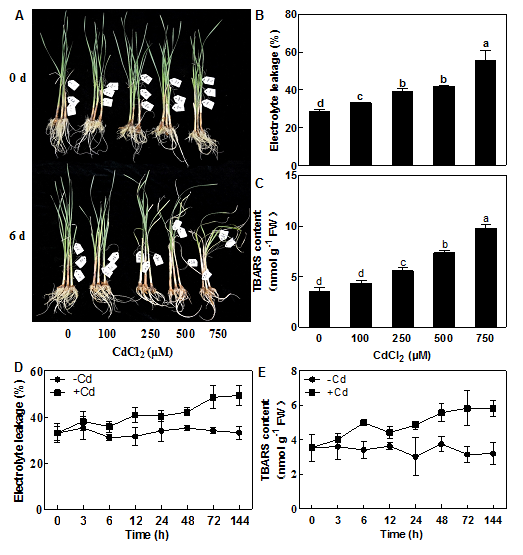


**Figure S1 Dose-dependent effects of CdCl_2_ on the *Typha angustifolia* seedlings growth (A), electrolyte leakage (B, D) and thiobarbituric acid reactive substance (TBARS) contents (C, E).** Seedlings were grown in nutrient solution in the presence of 0, 100, 250, 500, and 750 μM CdCl_2_ for 6 d. Photos showing response of *Typha angustifolia* seedlings to CdCl_2_ exposure were taken. Electrolyte leakage (B) and TBARS content (C) in roots were then analyzed. Time dependent changes of Electrolyte leakage (D) and TBARS content (E) under 250 μM CdCl_2_ exposure was also analyzed. Data are the means ± SE from three independent experiments. Six biological replicates were analyzed in each set of experiments. Within each set of experiments, bars with different letters are signiﬁcantly different at P < 0.05, according to Duncan’s multiple range test.
