# Supplementary material for: Transcriptome-Wide Identification of miRNAs and Their Targets from Typha angustifolia by RNA-Seq and Their Response to Cadmium Stress
Source: PLoS One. 2015 Apr 29;10(4):e0125462. doi: 10.1371/journal.pone.0125462 (PMC4414455; doi:10.1371/journal.pone.0125462)
Supplement: S2 Fig — For transcriptome analysis, data filtering included removing adaptors and low-quality reads from raw reads. Transcriptome de novo assembly was carried out with short reads assembling program Trinity. Unigenes were annotated with the databases of non-redundant protein (Nr), NCBI non-redundant nucleotide sequence (Nt), Swiss-Prot, Kyoto Encyclopedia of Genes and Genomes (KEGG), Cluster of Orthologous Groups of proteins (COG) and Gene Ontology (GO). For small RNA analysis, the 49 nt sequence tags from sequencing were gone through the data cleaning analysis first, which includeed getting rid of the low quality tags, 5’ adaptor contaminants from the 50 nt tags, to get credible clean tags. The standard analysis annotated the clean tags into different categories and taken those which could not be annotated to any category to predict the novel miRNA and seed edit of potential known miRNA. After getting miRNA result, target prediction for miRNAs and GO enrichment and KEGG pathway for target genes were analyzed. (DOC) [file pone.0125462.s002.doc]

Figure S2


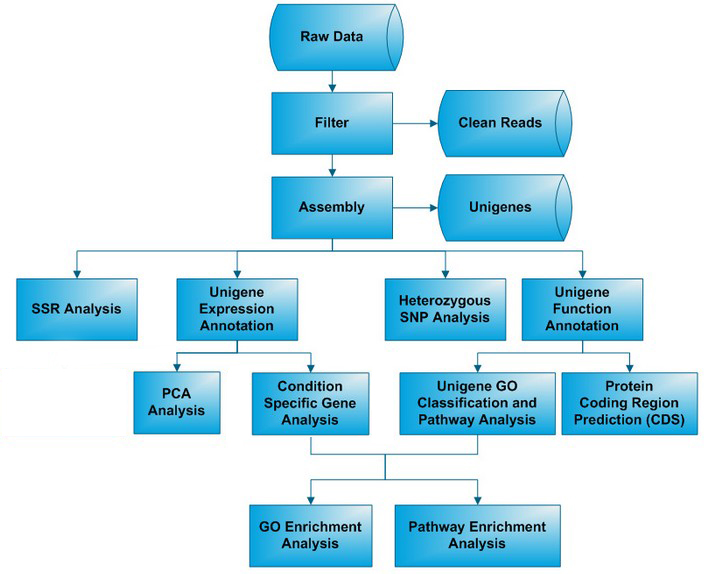


**A**


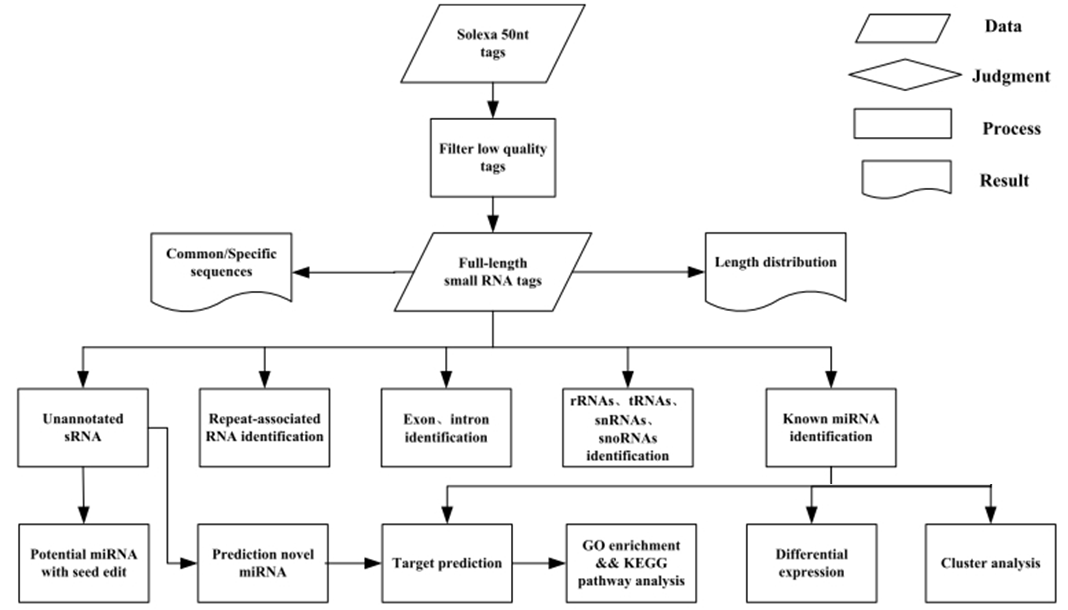


**B**

**Figure S2 Flow chart of the methodology adopted to transcriptome (A) and small RNA (B) analysis in *Typha angustifolia*.** For transcriptome analysis, data filtering included removing adaptors and low-quality reads from raw reads. Transcriptome *de novo* assembly was carried out with short reads assembling program Trinity. Unigenes were annotated with the databases of NR, NT, Swiss-prot, KEGG, COG and GO. For small RNA analysis, the 49 nt sequence tags from sequencing were gone through the data cleaning analysis first, which includeed getting rid of the low quality tags, 5’ adaptor contaminants from the 50 nt tags, to get credible clean tags. The standard analysis annotated the clean tags into different categories and taken those which could not be annotated to any category to predict the novel miRNA and seed edit of potential known miRNA. After getting miRNA result, target prediction for miRNAs and GO enrichment and KEGG pathway for target genes were analyzed.
