# Supplementary material for: Transcriptome-Wide Identification of miRNAs and Their Targets from Typha angustifolia by RNA-Seq and Their Response to Cadmium Stress
Source: PLoS One. 2015 Apr 29;10(4):e0125462. doi: 10.1371/journal.pone.0125462 (PMC4414455; doi:10.1371/journal.pone.0125462)
Supplement: S3 Fig — A, the horizontal coordinates are unigene or contig lengths and the vertical coordinates are numbers of unigenes. B, the horizontal coordinates are contig lengths and the vertical coordinates are numbers of contigs. (DOC) [file pone.0125462.s003.doc]

Figure S3


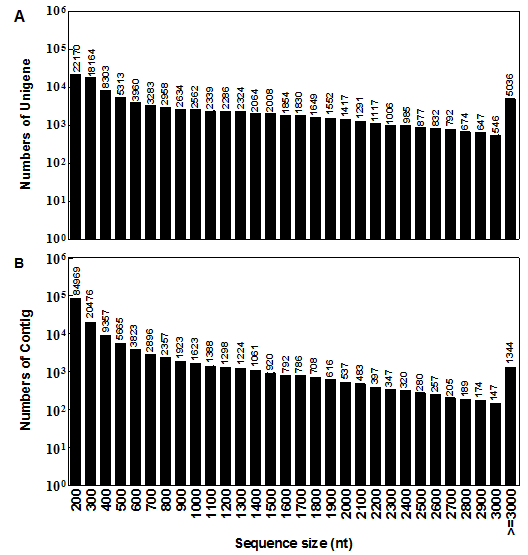


**Figure S3 Length distributions of *Typha angustifolia* unigenes (A) and contigs (B).** A, the horizontal coordinates are unigene or contig lengths and the vertical coordinates are numbers of unigenes. B, the horizontal coordinates are contig lengths and the vertical coordinates are numbers of contigs.
