# Supplementary material for: Transcriptome-Wide Identification of miRNAs and Their Targets from Typha angustifolia by RNA-Seq and Their Response to Cadmium Stress
Source: PLoS One. 2015 Apr 29;10(4):e0125462. doi: 10.1371/journal.pone.0125462 (PMC4414455; doi:10.1371/journal.pone.0125462)
Supplement: S4 Fig — Unigene sequences were firstly aligned to protein database NR by blastn, retrieving proteins with highest sequence similarity to the annotated protein in the database. Then counted and summarized annotation results. (DOC) [file pone.0125462.s004.doc]

Figure S4


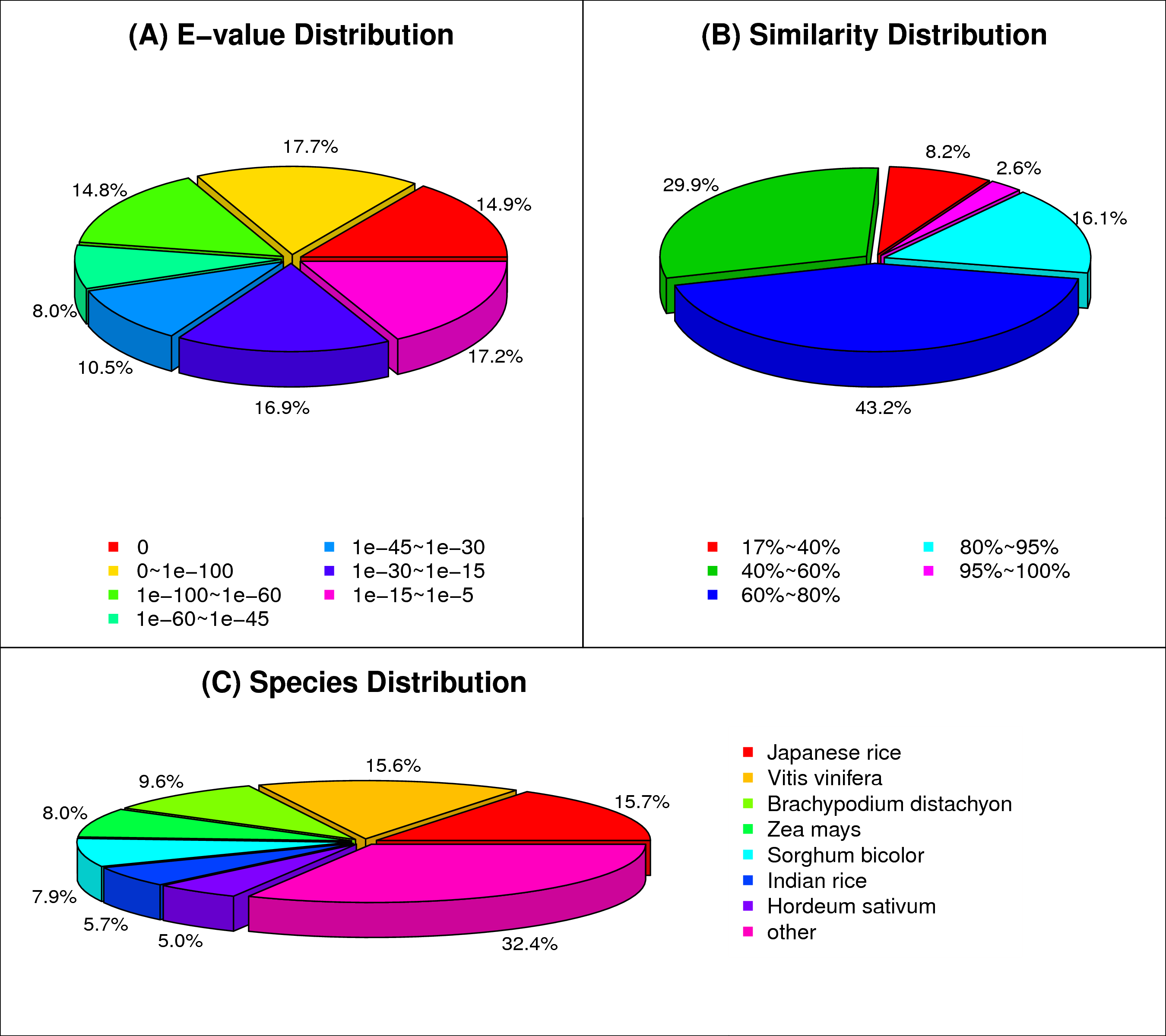


**Figure S4 The E-value distribution (A), similarity distribution (B) and the species distribution (C) of NR annotation.** Unigene sequences were firstly aligned to protein database NR by blastn, retrieving proteins with highest sequence similarity to the annotated protein in the database. Then counted and summarized annotation results.
