# Supplementary material for: Transcriptome-Wide Identification of miRNAs and Their Targets from Typha angustifolia by RNA-Seq and Their Response to Cadmium Stress
Source: PLoS One. 2015 Apr 29;10(4):e0125462. doi: 10.1371/journal.pone.0125462 (PMC4414455; doi:10.1371/journal.pone.0125462)
Supplement: S1 Table — (DOC) [file pone.0125462.s005.doc]

**Table S1** Criteria for analysis of conserved miRNAs, novel miRNAs, miRNA targets and expression level of miRNAs.

| **Analysis** | **Steps** |
| --- | --- |
| **Prediction of conserved miRNA** | 1. Considering the difference among species, align clean data to the miRNA precursor/mature miRNA of all plants/animals in miRBase allowing two mismatches and free gaps; 2. Choose the highest expression miRNA for each mature miRNA family which is regarded as a temporary miRNA database; 3. Align clean data to the above temporary miRNA database and the expression of miRNA is generated by summing the count of tags which can align to the temporary miRNA database within two mismatches. 4. We performed extensive comparisons against known miRNAs in other plant species to investigate the evolutionary conservation relationship of known miRNAs in *T. angustifolia* and other plants. |
| **Prediction of novel miRNAs** | 1. The tags which be used to predict novel miRNA are from the unannotated tags which can match to reference sequences, from the tags which can align to intron region and from the tags which can align to antisense exon region; 2. Those genes whose sequences and structures satisfied the two criteria, hairpin miRNAs can fold secondary structures and mature miRNAs are present in one arm of the hairpin precursors, will be considered as candidate miRNA genes; 3. The mature miRNA strand and its complementary strand (miRNA*) present 2-nucleotide 3' overhangs; 4. Hairpin precursors lack large internal loops or bulges; 5. The secondary structures of the hairpins are steady, with the free energy of hybridization lower than or equal to -18 kcal/mol; 6. The number of mature miRNA with predicted hairpin must be no fewer than 5 in the alignment result. |
| **Prediction of miRNA targets** | (1) No more than four mismatches between sRNA and target (G-U bases count as 0.5 mismatches); (2) No more than two adjacent mismatches in the miRNA/target duplex; (3) No adjacent mismatches in in positions 2-12 of the miRNA/target duplex (5' of miRNA); (4) No mismatches in positions 10-11 of miRNA/target duplex; (5) No more than 2.5 mismatches in positions 1-12 of the of the miRNA/target duplex (5' of miRNA); (6) Minimum free energy (MFE) of the miRNA/target duplex should be >= 75% of the MFE of the miRNA bound to it's perfect complement. |
| **Differential expression of miRNA** | (1) Normalize the expression of miRNA in two samples (control and treatment) to get the expression of transcript per million (TPM).  Normalization formula: Normalized expression = Actual miRNA count/Total count of clean reads*1000000; (2) Calculate fold-change and P-value from the normalized expression. Then generate the log2ratio plot and scatter plot. Fold-change formula: Fold_change=log_2_ (Cd/Ck). Cd/Ck means the normalized expression of miRNAs in Cd library was divided by the normalized expression in Ck library. After the Bayesian test, if the *P*-value given by this method was <0.01 and the fold-change in normalized sequence counts was more than one, a specific miRNA was considered to be differentially expressed. |
