# Supplementary material for: Transcriptome-Wide Identification of miRNAs and Their Targets from Typha angustifolia by RNA-Seq and Their Response to Cadmium Stress
Source: PLoS One. 2015 Apr 29;10(4):e0125462. doi: 10.1371/journal.pone.0125462 (PMC4414455; doi:10.1371/journal.pone.0125462)
Supplement: S2 Table — The primers of target genes were designed according to the respective sequence of contigs or Unigenes in transcriptome. The primers of miRNAs were designed according to the protocol of One Step PrimeScript miRNA cDNA Synthesis Kit (TaKaRa). (DOC) [file pone.0125462.s006.doc]

**Table S2** **The sequences of primers for real-time RT-PCR.**

| Primer name | Sequences (5’→3’) |
| --- | --- |
| *CL5275-F* | CATTTGGTTGGAAAGTATCTGGAC |
| *CL5275-R* | GAAAGGAAGGCTTATCTGGTGTT |
| *Unigene35908-F* | TTAGTTCCGAGCAAACAAGTC |
| *Unigene35908-R* | CCCGTGGTACTATGGAGGTG |
| *CL1727-F* | CTTAATTCATGTACGCCACCA |
| *CL1727-R* | TGCTCCAAATAATCTGACCAA |
| *Unigene11691-F* | TAAGGCTGTTAGTAGTTG |
| *Unigene11691-R* | AGGTAGTATCATCTCGTA |
| *CL7951-F* | ACCAAGCACCAGGACATGAT |
| *CL7951-R* | AAACCTTCGCCGCACTCT |
| *CL354-F* | AGAGTATTGAAAATGACAAGTGAAG |
| *CL354-R* | CTACATAAGATACTTTCTCTCCCTC |
| *CL3870-F* | TCATTGGAAACAAAACCTTGATTAG |
| *CL3870-R* | GCTGAAGGAAATAGGGACACG |
| *Unigene23735-F* | TCTACATTGCTTGTGCTCTTTG |
| *Unigene23735-R* | ATTTTCCATTAGTTGCCATTTC |
| *Unigene11164-F* | TTGGTATTCTCCGACATAGTGA |
| *Unigene11164-R* | GGGCTATTAGCAATTCCTGG |
| *Unigene11819-F* | ATGAGTTCAATAGGCATCCG |
| *Unigene11819-R* | CTGTTATTTTTTTCCCACCAA |
| *Unigene33298-F* | TTACAAGCCCAAGAAATAGCAG |
| *Unigene33298-R* | AGCATCCCTTATCTCCCTTAGTC |
| *Actin-F* | CTTCTCGGTTCGCTTTCTT |
| *Actin-R* | CTCCAACATAGGCATCTTTCTG |
| *miR1862e* | GCTAGATTTGTTTATTTTGGGACGG |
| *miR4414b* | CAGTGAATGATGCGGGAGGTAA |
| *miR529-3p* | GCCGCTGTACCCTCTCTCTTCTTC |
| *miR827* | GCGTTAGATGATCATCAGCAAACA |
| *novel_mir_10* | CGAGTGACCTGCGAACACATGA |
| *novel_mir_18* | GCGCTCCAATTAGATTGTCTTCTTT |
| *novel_mir_25* | TCAATGCGATTCCGTTGGAAT |
| *novel_mir_29* | GGGCGCAGCGGTTTATCG |
| *novel_mir_30* | GCTCACGTGTTGAAGATTGTCGA |
| *novel_mir_31* | CAGGCCTATGATGAACAGCAGAA |

The primers of target genes were designed according to the respective sequence of contigs or Unigenes in transcriptome. The primers of miRNAs were designed according to the protocol of One Step PrimeScript miRNA cDNA Synthesis Kit (TaKaRa).
