# Supplementary material for: Transcriptome-Wide Identification of miRNAs and Their Targets from Typha angustifolia by RNA-Seq and Their Response to Cadmium Stress
Source: PLoS One. 2015 Apr 29;10(4):e0125462. doi: 10.1371/journal.pone.0125462 (PMC4414455; doi:10.1371/journal.pone.0125462)
Supplement: S3 Table — Q20, base quality more than 20. N50 of contigs or unigenes were calculated by ordering all sequences, then adding the lengths from longest to shortest until the summed length exceeded 50% of the total length of all sequences. (DOC) [file pone.0125462.s007.doc]

**Table S3 Summary of Illumina transcriptome assembly for *T. angustifolia*.**

| Feature | Value |
| --- | --- |
| Total number of raw reads | 57,608,230 |
| Total number of clean reads | 54,321,956 |
| Total Clean Nucleotides (nt) | 4,888,976,040 |
| Q20 percentage | 98.25% |
| GC percentage | 47.47% |
| Total Number of contigs | 146,562 |
| Total Length of Contigs (nt) | 56,410,328 |
| Mean Length of contigs (nt) | 385 |
| N50 of contigs (nt) | 814 |
| Total Number of unigenes | 102,473 |
| Total Length of unigenes (nt) | 94,182,733 |
| Mean Length of unigenes (nt) | 919 |
| N50 of unigenes (nt) | 1802 |
| Distinct Clusters | 35,570 |
| Distinct Singletons | 66,903 |

Q20, base quality more than 20. N50 of contigs or unigenes were calculated by ordering all sequences, then adding the lengths from longest to shortest until the summed length exceeded 50% of the total length of all sequences.
