# Supplementary material for: Transcriptome-Wide Identification of miRNAs and Their Targets from Typha angustifolia by RNA-Seq and Their Response to Cadmium Stress
Source: PLoS One. 2015 Apr 29;10(4):e0125462. doi: 10.1371/journal.pone.0125462 (PMC4414455; doi:10.1371/journal.pone.0125462)
Supplement: S4 Table — The unigenes were annotated by aligning with the deposited ones in diverse protein databases including National Center for Biotechnology Information (NCBI) non-redundant protein (Nr) database, NCBI non-redundant nucleotide sequence (Nt) database, UniProt/Swiss-Prot, Kyoto Encyclopedia of Genes and Genomes (KEGG), Cluster of Orthologous Groups of proteins (COG) and Gene Ontology (GO). The overall functional annotation was summarized. (DOC) [file pone.0125462.s008.doc]

**Table S4 Summary of functional annotation of the *T. angustifolia* transcriptome.**

| Database | Number of annotated unigenes | Percentage |
| --- | --- | --- |
| NR | 57,962 | 56.56% |
| NT | 47,880 | 46.72% |
| SwissProt | 40,525 | 39.55% |
| KEGG | 38,058 | 37.14% |
| COG | 27,373 | 26.71% |
| GO | 44,307 | 43.24% |
| ALL | 60,303 | 58.85% |

The unigenes were annotated by aligning with the deposited ones in diverse protein databases including National Center for Biotechnology Information (NCBI) non-redundant protein (Nr) database, NCBI non-redundant nucleotide sequence (Nt) database, UniProt/Swiss-Prot, Kyoto Encyclopedia of Genes and Genomes (KEGG), Cluster of Orthologous Groups of proteins (COG) and Gene Ontology (GO). The overall functional annotation was summarized.
