# Supplementary material for: Transcriptome-Wide Identification of miRNAs and Their Targets from Typha angustifolia by RNA-Seq and Their Response to Cadmium Stress
Source: PLoS One. 2015 Apr 29;10(4):e0125462. doi: 10.1371/journal.pone.0125462 (PMC4414455; doi:10.1371/journal.pone.0125462)
Supplement: S6 Table — Statistics of small RNAs from CK and Cd-infected sample in both total and unique reads. rRNA, ribosome RNA. tRNA, transporter RNA. snRNA, small nuclear RNA. snoRNA, small nucleolar RNA. (DOC) [file pone.0125462.s010.doc]

**Supplementary Table S6 Statistics of small RNA sequences from CK and Cd libraries of the *Typha angustifolia*.**

|  | Total reads | Percent (%) | Unique reads | Percent (%) |
| --- | --- | --- | --- | --- |
| CK |  |  |  |  |
| Raw reads | 12,128,241 |  |  |  |
| Clean reads (18-30 nt sRNA) | 11,412,343 | 100% | 2,654,641 | 100% |
| miRNA | 1,836,897 | 16.1% | 37,645 | 1.42% |
| rRNA/tRNA/snRNA/snoRNA | 3,555,831 | 31.16 | 306,282 | 11.54% |
| Un-annotation | 6,019,615 | 52.75% | 2,310,714 | 87.04% |
| Cd |  |  |  |  |
| Raw reads | 12,392,054 |  |  |  |
| Clean reads (18-30 nt sRNA) | 11,799,855 | 100% | 2,897,906 | 100% |
| miRNA | 1,813,553 | 15.37% | 41,002 | 1.41% |
| rRNA/tRNA/snRNA/snoRNA | 3,524,411 | 29.86% | 350,363 | 12.09% |
| Un-annotation | 6,461,891 | 54.76% | 2,506,541 | 86.49% |

Statistics of small RNAs from CK and Cd-infected sample in both total and unique reads. rRNA, ribosome RNA. tRNA, transporter RNA. snRNA, small nuclear RNA. snoRNA, small nucleolar RNA.
