# Supplementary material for: Transcriptome-Wide Identification of miRNAs and Their Targets from Typha angustifolia by RNA-Seq and Their Response to Cadmium Stress
Source: PLoS One. 2015 Apr 29;10(4):e0125462. doi: 10.1371/journal.pone.0125462 (PMC4414455; doi:10.1371/journal.pone.0125462)
Supplement: S7 Table — Identified T. angustifolia conserved miRNAs and their reads in CK and Cd libraries. The identification was performed by selecting homologies to temporary miRNA database of T. angustifolia within two mismatches. (DOC) [file pone.0125462.s011.doc]

**Supplementary Table S7 Conserved miRNAs in *Typha angustifolia*.**

| **miRNA family** | **miRNA name** | **Sequence in the Typha angustifolia (5’-3’)** | **CK reads** | **Cd reads** |
| --- | --- | --- | --- | --- |
| miR156 | miR156a | TGACAGAAGAGAGTGAGCAC | 229669 | 212927 |
|  | miR156c-3p | TGCTCACTTCTCTATCTGTCAGA | 277 | 334 |
| miR157 | miR157d-3p | GCTCTCTATGCTTCTGTCATC | 89 | 112 |
| miR159 | miR159a | TTTGGATTGAAGGGAGCTCTA | 1539 | 1351 |
| miR160 | miR160a | TGCCTGGCTCCCTGTATGCCA | 194 | 196 |
|  | miR160a-3p | GCGTGCAAGGAGCCAAGCATG | 185 | 300 |
| miR162 | miR162 | TCGATAAACCTCTGCATCCGG | 2100 | 1898 |
| miR164 | miR164a | TGGAGAAGCAGGGCACGTGCA | 15757 | 12575 |
|  | miR164a-3p | CATGTGCCCATCTTCTCCACC | 35 | 58 |
| miR165 | miR165a | TCGGACCAGGCTTCATACCCC | 478 | 440 |
|  | miR165a-3p | TCGGACCAGGCTTTCCCC | 521 | 586 |
| miR166 | miR166a | TCGGACCAGGCTTCATTCCCC | 190846 | 189088 |
|  | miR166g-3p | TCGGACCAGGCTTCATTCCTC | 10327 | 6595 |
| miR167 | miR167a | TGAAGCTGCCAGCATGATCTGA | 163547 | 143139 |
|  | miR167f-3p | AGATCATGTGGCAGTTTCATC | 588 | 723 |
| miR168 | miR168a | TCGCTTGGTGCAGGTCGGGAA | 70388 | 64436 |
|  | miR168a-3p | CCCGCCTTGCATCAACTGAAT | 405 | 522 |
| miR169 | miR169b | CAGCCAAGGATGACTTGCCGG | 2592 | 2191 |
|  | miR169c-3p | GGCAAGTTTGTCCTTGGCTAC | 82 | 73 |
| miR170 | miR170 | TGATTGAGCCGTGTCAATATC | 238 | 196 |
| miR171 | miR171 | TTGAGCCGCGTCAATATCTCC | 553 | 410 |
|  | miR171b-3p | CGAGCCGAACCAATATCACTC | 155 | 224 |
| miR172 | miR172a | AGAATCTTGATGATGCTGCAT | 3909 | 3058 |
| miR319 | miR319a | TTGGACTGAAGGGAGCTCCCT | 107 | 80 |
|  | miR319a-3p | TTGGACTGAAGGGAGCTCCC | 73 | 50 |
| miR390 | miR390a | AAGCTCAGGAGGGATAGCGCC | 393 | 237 |
|  | miR390a-3p | CGCTATCTATCCTGAGTTTCA | 15 | 9 |
| miR391 | miR391 | CTCGCAGGAGAGATGACGCCG | 697 | 725 |
| miR393 | miR393h | TTCCAAAGGGATCGCATTGAT | 973 | 731 |
| miR394 | miR394a | TTGGCATTCTGTCCACCTCC | 24 | 34 |
|  | miR394b-3p | AGGTGGGGATGACGTCAAGT | 5236 | 3731 |
| miR395 | miR395a-5p | GTTCTCTCCAAGCACTTCATT | 12 | 23 |
| miR396 | miR396c-3p | GGTCAAGAAAGCTGTGGGAAG | 437 | 298 |
|  | miR396e-5p | TCCACAGGCTTTCTTGAACAG | 5169 | 3489 |
| miR397 | miR397a | TCATTGAGTGCAGCGTTGATG | 126 | 87 |
| miR408 | miR408b | ACAGGGATGAGATAGAGCATG | 4405 | 3130 |
| miR444 | miR444a | TGCTGCCTCAAGCTTGCTGCC | 941 | 1094 |
| miR479 | miR479 | CGTGGTATTGGTTCGGTTCATC | 39 | 62 |
| miR482 | miR482b-3p | TTGCCAATACCGTCCATGCCGA | 1000 | 1052 |
| miR528 | miR528-5p | TGGAAGGGGCATGCAGAGGAG | 12166 | 10235 |
| miR529 | miR529-3p | GCTGTACCCTCTCTCTTCTTC | 64 | 31 |
|  | miR529-5p | AGAAGAGAGAGAGTACAGCCT | 2011 | 1073 |
| miR530 | miR530a | TGCATTTGCACCTGCACCTTT | 341 | 282 |
| miR533 | miR533e | CACACAGGTGCTGCATGGCTGTC | 4680 | 4797 |
| miR535 | miR535d | TGACGATGAGAGAGAGCACGC | 128168 | 100292 |
|  | miR535-3p | GTGCTCTCTCTCGTTGTCACT | 56 | 65 |
| miR812 | miR812g | AAGACGGATGATTAAAGTTGGACA | 33 | 26 |
| miR827 | miR827 | TTAGATGATCATCAGCAAACA | 244 | 114 |
| miR845 | miR845a | TGGCTGTGATACCAAGTTGAA | 45 | 34 |
| miR858 | miR858b | TTCGTTGTCTGTTCGACCTTG | 7 | 9 |
| miR894 | miR894 | GTTTCACGTCGGGTTCACCA | 46424 | 56951 |
| miR902 | miR902c-3p | ACGAAAGTCGGTCATAGT | 2967 | 3193 |
| miR916 | miR916 | CCGAAGGTCGTCGGTTCAAATCC | 873 | 888 |
| miR952 | miR952b | AACGAGGATCCATTGGAG | 3493 | 4894 |
| miR1077 | miR1077-5p | TTGAAGTGTTCGGATCGCGGC | 33739 | 41403 |
| miR1092 | miR1092 | TGACAGGAAGTGCAGTAGTTT | 27 | 31 |
| miR1172 | miR1172.2 | TCGGACTGAGACGCAGTGA | 128 | 190 |
| miR1223 | miR1223b | TTATTGTAGCATACACCTCTA | 118 | 113 |
| miR1310 | miR1310 | GAGGCATCGGGGGCGCAACGCCCT | 2451 | 2874 |
| miR1432 | miR1432-5p | TCAGGAGAGATGACACCCGCG | 3223 | 1857 |
| miR1520 | miR1520d | ATCAGAACTGGTACGGACAA | 69253 | 71936 |
| miR1850 | miR1850.1 | TGGAAAGTTGGGAGATTGGGG | 16 | 19 |
| miR1854 | miR1854-5p | TGTGAAATTAGTAGAATAGGA | 96 | 85 |
| miR1861 | miR1861c | TGATCTTTGTAGAAGAACTGTG | 15438 | 11874 |
| miR1862 | miR1862e | CTAGATTTGTTTATTTTGGGACGG | 28 | 14 |
| miR1870 | miR1870-5p | TGCTGAATTAGACCTAGTGGGCAT | 7 | 9 |
| miR2089 | miR2089-3p | AGGATTGGCTCTAAAGGTAGAA | 274 | 310 |
| miR2111 | miR2111c | TCATCTCATCCTGGGGCTGTA | 2750 | 3654 |
| miR2118 | miR2118d | TTCCGATCCCTCCCATGCCGA | 1488 | 1618 |
| miR2199 | miR2199 | TGATAACTCGACGGATCGC | 50140 | 43292 |
| miR2667 | miR2667a | TCTTTGATCTGAGCGGTAGC | 106 | 181 |
| miR2867 | miR2867-3p | CCAGGACGTTGATAGGCA | 3737 | 3453 |
| miR2916 | miR2916 | GGGGCTCGAAGACGATCAGAT | 14847 | 12588 |
| miR4376 | miR4376 | TCAGGAGAGATGACACCCGT | 51 | 39 |
| miR4414 | miR4414b | AGTGAATGATGCGGGAGGTAA | 314 | 94 |
| miR4995 | miR4995 | TAGGCAGTGGCTTGGTTAAGGG | 92 | 175 |
| miR5029 | miR5029 | TATGAAAGACGAACAACTGCAAA | 15370 | 15872 |
| miR5054 | miR5054 | TTTCCCACGGACGGCGCCA | 7630 | 8484 |
| miR5059 | miR5059 | TCGTGCCTGGGCAGCACCACCA | 5037 | 6832 |
| miR5070 | miR5070 | AGCTAGAGTACGGTAGAGGGT | 2397 | 1878 |
| miR5072 | miR5072 | CGTTCCCCAGCAGAGTCGCCA | 590 | 808 |
| miR5077 | miR5077 | TTCACGTCGGGTTCACCA | 18557 | 19033 |
| miR5083 | miR5083 | AGACTACAATTATCTGATCA | 19 | 21 |
| miR5139 | miR5139 | AACCTGGCTCTGATACCA | 939 | 939 |
| miR5152 | miR5152-3p | AGTCCTGCTATACCCACCA | 92 | 156 |
| miR5179 | miR5179 | TTTTGCTCAAGACCGCGCAAC | 64 | 41 |
| miR5225 | miR5225a | TTTGTCAGGAGAGATGACAC | 74 | 61 |
| miR5228 | miR5228 | TTGGTGTAGATCTTGATGGTA | 1134 | 1132 |
| miR5265 | miR5265 | AAGTGATGGTGGAAGGGTTAT | 1100 | 645 |
| miR5293 | miR5293 | GATGAAGACGAGGAAGAGAAGAGA | 72 | 50 |
| miR5301 | miR5301 | TGTGGGTAGGGGTGAAAGGCT | 1779 | 1431 |
| miR5368 | miR5368 | AGGGACAGTCTCAGGTAGACA | 372 | 760 |
| miR5386 | miR5386 | CGTCGGCTGTCGGCGGACTG | 7740 | 12534 |
| miR5492 | miR5492 | AGAAGGAGGAAAGAATGGGTT | 102 | 70 |
| miR5512 | miR5512a | TCGGATATGGTCGAATGGTAAAA | 11951 | 11203 |
| miR5641 | miR5641 | TAGGAAGAAGATGATGGAACT | 25 | 31 |
| miR5656 | miR5656 | TGAAGTAGAGACTGGGTTA | 635 | 738 |
| miR5671 | miR5671 | CATGGTGGTGACGGGTGAC | 5557 | 4205 |
| miR5794 | miR5794 | TGAGGAATCACTAGTAGTCGT | 78 | 72 |
| miR5800 | miR5800 | CTCGGATTCGGAACGGCTC | 50935 | 49675 |
| miR5813 | miR5813 | ACAGCAGGACGGTGGTCATGGA | 30980 | 28387 |
| miR6135 | miR6135k | CGTGTCGTGGTGTAGTTGGT | 133944 | 133551 |
| miR6171 | miR6171 | TCTGTGGATGGCTGAAGGCTT | 150 | 86 |
| miR6205 | miR6205 | AGGATGTTTGGATAGATGTAT | 225 | 288 |
| miR6281 | miR6281 | TTAGAGATAGAGGAGTGCT | 27 | 14 |
| miR6300 | miR6300 | GTCGTTGTAGTATAGTGGT | 14480 | 15874 |
| miR6441 | miR6441 | AATTGACGGAAGAACACAA | 7690 | 6818 |
| miR6463 | miR6463 | TGGATGATACGTGGCAACA | 44 | 26 |
| miR6478 | miR6478 | CCGACCTTAGCTCAGTTGGTA | 2034 | 1705 |
| miR7533 | miR7533a | GAGGCGATCGAGAGAACTG | 176 | 100 |
| miR7545 | miR7545 | TTGAAGAAATTAGAGTGCT | 743 | 894 |
| miR7732 | miR7732-3p | GTAGAGATCGGGAGGAACA | 2756 | 4501 |
| miR7767 | miR7767-5p | CCCCAAGATGAGTGCTCTCC | 419 | 718 |
| miR8155 | miR8155 | TAACCTGGCTCTGATACCA | 988 | 1009 |

Identified *T. angustifolia* conserved miRNAs and their reads in CK and Cd libraries. The identification was performed by selecting homologies to temporary miRNA database of *T. angustifolia* within two mismatches.
